# Multimodal Imaging in the Management of Choroidal Neovascularization Secondary to Central Serous Chorioretinopathy

**DOI:** 10.3390/jcm9061934

**Published:** 2020-06-21

**Authors:** Ahmed M. Hagag, Shruti Chandra, Hagar Khalid, Ali Lamin, Pearse A. Keane, Andrew J. Lotery, Sobha Sivaprasad

**Affiliations:** 1 Moorfields Eye Hospital NHS Foundation Trust, London EC1V 2PD, UK; ahmed.hagag@nhs.net (A.M.H.); shruti.chandra@nhs.net (S.C.); hagar.khalid@nhs.net (H.K.); a.lamin@nhs.net (A.L.); pearse.keane1@nhs.net (P.A.K.); 2 UCL Institute of Ophthalmology, London EC1V 9EL, UK; 3 Faculty of Medicine, University of Southampton, Southampton SO17 1BJ, UK; A.J.Lotery@soton.ac.uk

**Keywords:** central serous chorioretinopathy, optical coherence tomography angiography, choroidal neovascularization, anti-VEGF, bevacizumab, suspected CNV, presumed CNV

## Abstract

The diagnosis and treatment of choroidal neovascularization (CNV) in eyes with chronic central serous chorioretinopathy (CSCR) can be challenging. The purpose of this study was to classify eyes with suspected CNV using multimodal imaging. The effect of intravitreal anti-vascular endothelial growth factor (VEGF) was assessed and compared to controls. This retrospective study included chronic CSCR patients with suspected secondary CNV who received intravitreal bevacizumab. Eyes were divided into “definite CNV” and “no CNV” based on optical coherence tomography angiography (OCTA). Eyes that did not undergo OCTA imaging were considered as “presumed CNV”. One-year outcome in visual acuity (VA) and central foveal thickness (CFT) were investigated and compared to non-treated control patients to assess the response to anti-VEGF. Logistic regression analysis was used to explore predictive biomarkers of CNV detection and improvement after anti-VEGF. Ninety-two eyes with chronic CSCR from 88 participants were included in this study. Sixty-one eyes received bevacizumab and 31 eyes were non-treated control subjects. The presence of subretinal hyperreflective material (SHRM) and shallow irregular retinal pigment epithelium (RPE) elevation (SIRE) with sub-RPE hyperreflectivity on OCT was associated with a significantly increased risk of detecting CNV on OCTA. Intravitreal anti-VEGF caused significant functional and anatomical improvement in patients with neovascular CSCR as compared to non-treated eyes. In contrast, VA and CFT changes were not significantly different between treated and non-treated CSCR with no evidence of CNV on OCTA. No clinical or anatomical biomarkers were found to be associated with response to treatment. In conclusion, OCTA should be used to confirm the presence CNV in suspected chronic CSCR patients. Intravitreal anti-VEGF treatment resulted in a significantly better one-year outcome in patients with definitive OCTA evidence of CNV.

## 1. Introduction

Central serous chorioretinopathy (CSCR) is a chorioretinal disease typically characterized by serous subretinal detachment associated with retinal pigment epithelium (RPE) and/or choroidal abnormalities [1,2]. It is the fourth most common acquired retinal disease with risk of visual impairment [3]. Patients with macula-involving acute CSCR usually present with blurred vision, metamorphopsia, relative central scotoma, or decreased contrast sensitivity due to accumulation of subretinal fluid (SRF) with/without focal pigment epithelial detachment (PED) evident on optical coherence tomography (OCT). The majority of acute CSCR cases are self-limiting with good visual outcome [4]. However, recurrences can occur in 50% of the affected individuals [5]. Chronic CSCR, although assumed to progress from acute CSCR, is likely to be an independent retinal pigment epitheliopathy secondary to choroidal abnormalities [6,7].

Choroidal neovascularization (CNV) is not an uncommon complication of chronic CSCR, with reported incidence of 2–18% of patients [8,9,10]. The development of CNV has been associated with significant visual loss in several retinal diseases [9,11,12,13]. Early detection and effective treatment of CNV can improve visual prognosis and prevent irreversible retinal damage [14]. Intravitreal anti-vascular endothelial growth factor (anti-VEGF) agents, especially bevacizumab (Avastin^®^), is the most widely used treatment option for CSCR related choroidal neovascularization [15].

However, the diagnosis of CNV in chronic CSCR patients can be challenging. Persistent SRF has often triggered multimodal imaging to rule out CNV [16]. While the double layer sign seen on OCT and active leakage on fluorescein angiography (FA) are signs of CNV, these are difficult to interpret in an eye with significant retinal pigment epithelial changes. More recently, OCT angiography (OCTA) has been introduced into clinical practice [17]. While the presence of CNV on OCTA has provided confidence in the definitive diagnosis of CNV, the absence of a visible CNV network on OCTA cannot rule out a CNV. In these circumstances, some clinicians prefer watchful waiting for spontaneous resolution of SRF, while others initiate anti-VEGF based on empirical decisions of “suspected” CNV. As the SRF in chronic CSCR can wax and wane, the treatment response to anti-VEGF in such scenarios is unclear.

Therefore, this study aimed at classifying the presence and absence of CNV in eyes with chronic CSCR based on multimodal imaging and assessed the effect of intravitreal bevacizumab in definitive versus presumed CNV and compared these to controls where the diagnosis of CNV was uncertain.

## 2. Methods

### 2.1. Study Participants and Clinical Data

**Figure 1:**
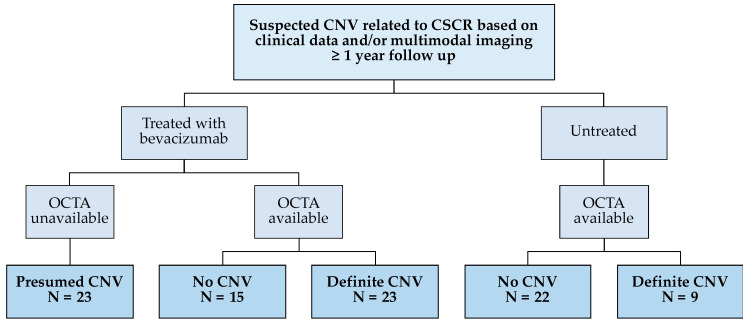
Flowchart of study groups. CNV, choroidal neovascularization; CSCR, central serous chorioretinopathy; OCTA, optical coherence tomography angiography.

The study was approved by the Moorfields Clinical Effectiveness Department (CA18/MR/22-197). Patients with a suspected diagnosis of CNV secondary to chronic CSCR were identified retrospectively from Moorfields Eye Hospital database. The diagnosis of “suspected” CNV was taken during routine clinical visits based on clinical data as well as spectral domain (SD) OCT and FA findings. Eyes that received bevacizumab and had at least one-year follow-up were included in this study. The anti-VEGF treatment was initiated as either three monthly loading doses or one injection followed directly by a “pro-re-nata” (PRN) regimen. No widely-accepted treatment protocol is available for using anti-VEGF in CSCR. Thus, the decision for choosing treatment regimen was based on the treating physician’s clinical judgement and individual preference. Non-treated chronic CSCR patients where CNV could not be established on multimodal imaging were also included as controls. All patients underwent SD-OCT scans during their follow-up clinical visits, and a subset of the included patients had OCTA imaging. *En face* images and B-scans from OCTA data were retrospectively reviewed by an expert grader to confirm the presence of CNV. Eyes were divided based on evidence of CNV on OCTA (Figure 1). The clinician’s decision of treatment was based on clinical data including history and the available multimodal imaging findings, which did not necessarily include OCTA. We retrospectively classified all eyes with “suspected” CNV by OCTA into “definite CNV” and “no CNV”. Thus, our classification was independent of the initial clinical diagnosis and treatment (Figure 1).

Clinic based visual acuity (VA) at baseline and one-year follow-up visits were reported. Gaining 1 line on Snellen’s chart or 5 letters on an early treatment diabetic retinopathy study (ETDRS) chart were considered as improvement in VA [18]. All VA measurements were converted to ETDRS for statistical analysis. Demographic data, duration of CSCR, history of steroid use (systemic or topical) and history of previous photodynamic therapy (PDT) or injection treatments were collected from clinical notes. Patients who underwent any ophthalmic surgical or medical intervention during or within six months prior to the one-year investigation period were not included in the study.

### 2.2. Multimodal Imaging

**Figure 2:**
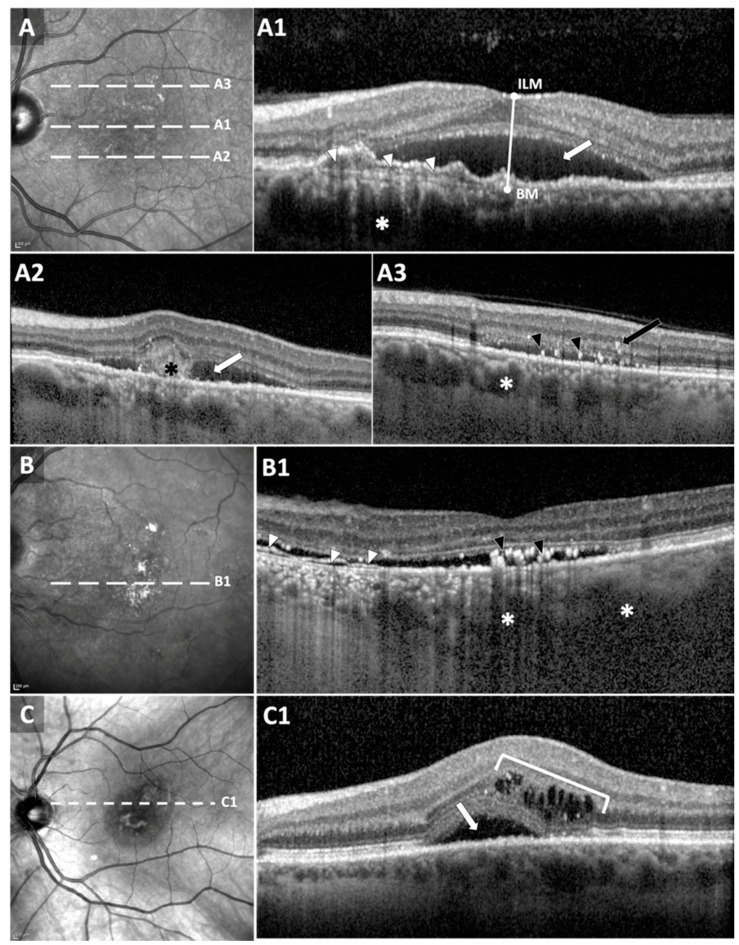
Quantitative and qualitative optical coherence tomography (OCT) biomarkers. (**A**) *En face* structural OCT in a “definite CNV” patient. White dashed lines correspond to OCT B-scans in (**A1**–**A3**). Central foveal thickness was measured between inner limiting membrane (ILM) and Bruch’s membrane (BM). Baseline anatomical biomarkers were detected from OCT B-scans: subretinal fluid (SRF, white arrows in (**A1**,**A2**)), shallow irregular RPE elevation (SIRE) with sub-RPE hyperreflectivity (white arrow heads in (**A1**)) and pachychoroid with dilated vessels (white asterisks). Subretinal hyperreflective material (black asterisk in (**A2**)) and intraretinal (IHRF, black arrow) subretinal hyperreflective foci (SHRF, black arrow heads) were also detected. (**B**,**C**) *En face* structural OCT in “no CNV” patients. White dashed lines correspond to B-scans in (**B1**,**C1**). White arrow heads in (**B1**) correspond to SIRE with sub-RPE hyporeflectivity. SHRF (black arrow heads) and pachychoroid (white asterisks) can be also detected. B-scan in (**C1**) exhibited SRF and intraretinal cysts.

The SD-OCT scans of the macula were acquired using the commercially available 3D OCT-2000 (Topcon, Tokyo, Japan) or Spectralis (Heidelberg Engineering, Heidelberg, Germany). Scans at baseline (day of the first injection) and one-year visits were reviewed for the purpose of this study. Central foveal thickness (CFT) was measured between inner limiting membrane (ILM) and Bruch’s membrane (BM) (Figure 2). A decrease of 20% in one-year CFT as compared to baseline was considered as anatomical improvement [18]. OCT scans were qualitatively reviewed at baseline to detect pathological signs including shallow irregular RPE elevation (SIRE), SRF, subretinal hyperreflective material (SHRM), intraretinal hyperreflective foci (IHRF), subretinal hyperreflective foci (SHRF), intraretinal cystic changes (IRC) and pachychoroid (Figure 2). SIRE was also identified as hyperreflective or hyporeflective depending on sub-RPE reflectivity (Figure 2). OCT grading was performed independently by two graders, and discrepancies were resolved by consensus.

Fluorescein angiography was reviewed by two graders to detect neovascularization. CNV was defined by the presence of early hyperfluorescence that increased in late frames or an area of late leakage. However, many cases were not straightforward due to the presence of RPE window defect in chronic CSCR. Additionally, the source of leak might not be known whether it was CNV or the CSCR itself.

**Figure 3:**
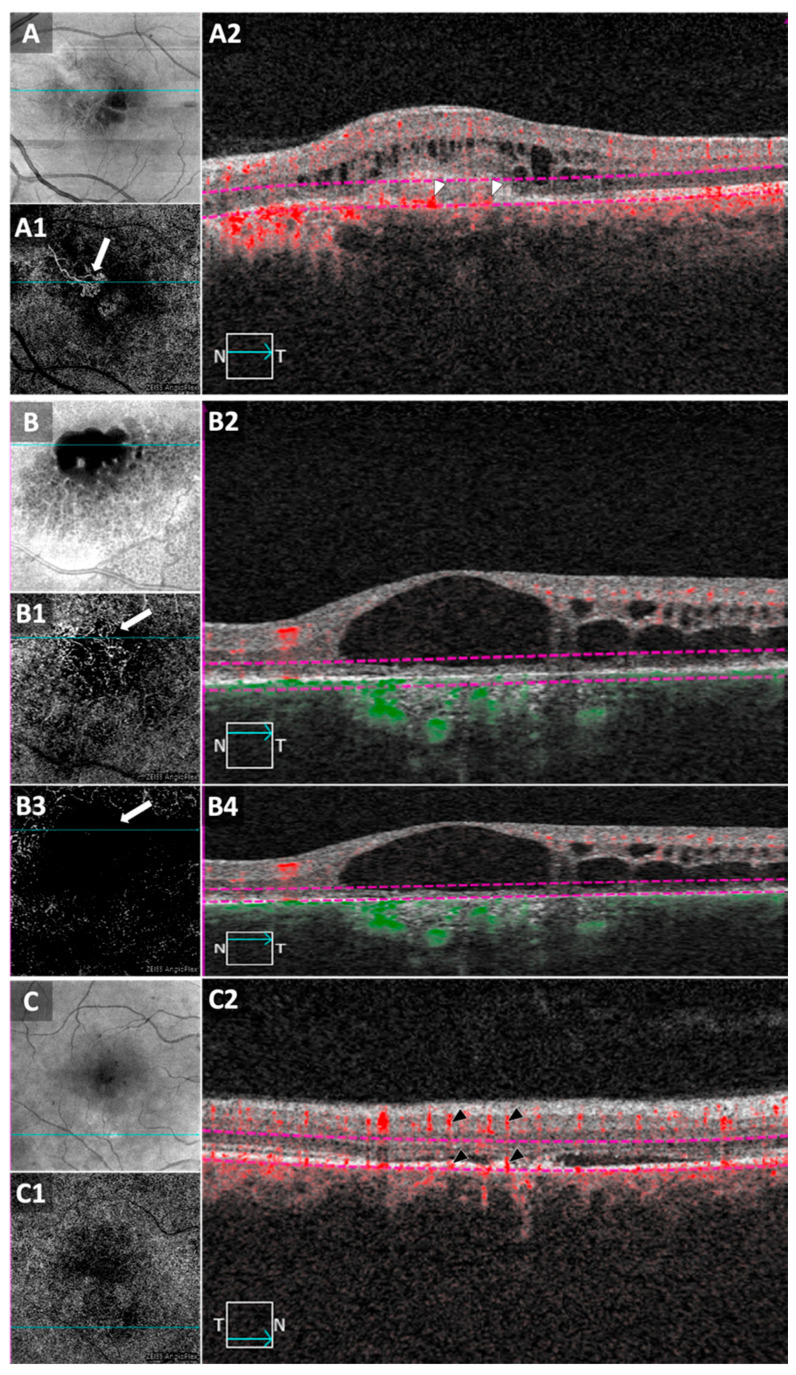
Optical coherence tomography angiography (OCTA) in patients with chronic central serous chorioretinopathy (CSCR). (**A**) *En face* structural OCT image from a 6 mm × 6 mm macular scan. (**A1**) *En face* OCT angiogram of the outer retina showing choroidal neovascularization (CNV, white arrow). Cyan lines in (**A**,**A1**) correspond to the B-scan in (**A2**). Red flow signal can be detected above Bruch’s membrane (BM). Purple dashed lines in (**A2**) represent the slab boundaries for the *en face* OCTA in (**A1**). (**B**) *En face* structural OCT from a 3 mm × 3 mm macular scan. (**B1**) *En face* OCTA of the “outer retina to choriocapillaris” (ORCC) slab displays flow signal that can resemble CNV (white arrow). Slab boundaries are overlaid in purple on the OCT/OCTA B-scan in (**B2**). B-scan shows RPE and CC atrophy with choroidal hypertransmission. No flow signal can be detected above BM. Vessels appearing in (**B1**) are most likely large choroidal vessels, not CNV. *En face* OCTA in (**B3**) corresponds to outer retinal slab (boundaries in (**B4**)), without including flow signal from the choroid. No suspicious vessels are seen in (**B3**), confirming the absence of CNV in this area. (**C**,**C1**) *En face* structural OCT and OCTA images, respectively, from 6 mm × 6 mm macular scan in a patient suspected for CNV. Cyan lines correspond to B-scan in (**C2**). Flow signal was observed at the area of RPE/outer retinal abnormalities (bottom black arrow heads). However, the flow signal is most likely to be projection artifacts from retinal vasculature (top black arrow heads) and not CNV. These examples demonstrate the importance of reviewing both *en face* and cross-sectional B-scans when interpreting OCTA images.

The OCTA scans were performed using Cirrus HD-OCT 5000 with AngioPlex software (Carl Zeiss Meditec, Inc., Dublin, CA, USA). Volumetric 3 mm × 3 mm or 6 mm × 6 mm scans were acquired, consisting of 175 B-scans. *En face* angiograms as well as cross-sectional OCT/OCTA images were carefully reviewed by two expert graders to confirm the presence/absence of CNV (Figure 3). Discrepancies were resolved by consensus.

### 2.3. Statistical Analysis

Statistical analysis was performed on SPSS v 26 (IBM, Armonk, New York, USA) and Microsoft Excel 2013 (Microsoft, Redmond, Washington, USA). The normality of data distribution was checked using Kolmogorov–Smirnoff [19] and the use of either parametric or non-parametric tests was decided accordingly. Continuous data are presented as mean ± standard deviation (SD), while categorical data are presented as frequency and/or percentage. Cohen’s kappa coefficient was used to assess inter-grader reliability for OCT, FA and OCTA. Mann–Whitney U test was used to compare means of two groups. Kruskal–Wallis test was used to compare between more than two groups. When a statistically significant difference was detected between groups, the Dunn post hoc test was used to explore pairwise comparisons. Longitudinal functional and structural changes within groups were assessed using Wilcoxon signed-ranks test. Pearson’s Chi-square test was used to investigate the differences in categorical variables. Adjusted standardized residuals (z-score) was used as a post-hoc analysis for Chi-square test [20]. Cells with absolute adjusted residuals larger than 1.96 were considered statistically significant. Univariable logistic regression analysis was used to explore clinical and anatomical predictive factors for the detection of CNV in suspected chronic CSCR patients. Clinically-relevant variables as well as variables with a *p* value < 0.2 were included in a multivariable model to detect independent CNV predictive biomarkers in chronic CSCR. Odds ratio (OR) with 95% confidence interval (CI) are reported in this paper. A similar approach was used to identify predictors for the response to anti-VEGF treatment. *p* values < 0.05 were considered statistically significant. Whenever needed, the Holm–Bonferroni method was employed to adjust for multiple comparisons [21].

## 3. Results

### 3.1. Participant Demographics and Clinical Data

Ninety-two eyes with chronic CSCR from 88 participants were included in this study. Sixty-one eyes received bevacizumab and 31 eyes were non-treated control subjects. In the treated group, 23 eyes did not undergo OCTA imaging and were considered to have “presumed CNV” based on OCT and FA. Twenty-three and 15 eyes were included in the “definite CNV” and “no CNV” groups, respectively, based on OCTA. Similarly, in the control group, 9 and 22 eyes were included in the “definite CNV” and “no CNV” non-treated groups based on OCTA. Clinical and demographic data are summarized in Table 1.

No statistically significant differences were detected between groups in terms of age and mean duration of CSCR (*p* = 0.06 and 0.65, respectively, Kruskal–Wallis test). However, treated “presumed CNV” eyes had relatively worse baseline VA as compared to other groups (*p* = 0.001, Kruskal–Wallis test, and *p* ≤ 0.04, Dunn post hoc tests). Meanwhile, the non-treated “definite CNV” control group had significantly thinner baseline CFT (*p* < 0.001, Kruskal–Wallis test, and *p* ≤ 0.01, Dunn post hoc tests) (Table 2).

### 3.2. Inter-Grader Reliability of OCT, FA and OCTA

The identification of OCT biomarkers showed some degree of inter-grader variability. The agreement between graders ranged from poor for IHRF, pachychoroid, SRF and SHRF (kappa = 0.15–0.39) to fair agreement in SIRE, SHRM and IRC (kappa = 0.56–0.69). The reliability of CNV detection using OCTA was significantly higher than FA in our cohort (kappa = 0.91 and 0.14, respectively, *p* < 0.001).

### 3.3. OCT Biomarkers Predicting Presence of CNV

Univariable logistic regression analyses revealed that older patients, female gender, hyperreflective SIRE and SHRM associated with significantly higher risk for CNV detection on OCTA. In contrast, SIRE with sub-RPE hyporeflectivity associated with the absence of CNV. The multivariable model identified hyperreflective SIRE (OR, 13.8, 95% CI, 3.0–63.7, *p* = 0.001) and SHRM (OR, 5.7, 95% CI, 1.3–24.9, *p* = 0.02) to be independent predictive factors for the presence of CNV on OCTA.

### 3.4. One-Year Changes in VA and CFT within Groups

Overall, this cohort of chronic CSCR patients showed significantly better VA and thinner CFT after one-year of initiating anti-VEGF injections (*p* < 0.001, Wilcoxon signed-ranks tests). However, when investigating each group separately, the significance was found to be originating from the “definite CNV” group (*p* = 0.03) with 96% of patients showing improvement in either VA or CFT (Table 2). In contrast, only 67% and 70% of patients in “no CNV” and “presumed CNV” groups, respectively, showed functional or structural improvement (Table 2). Mean ETDRS and CFT measurements at baseline and after one year were statistically equivalent within both groups (*p* > 0.09, Wilcoxon signed-ranks tests).

Similarly, no statistically significant differences in VA and CFT were observed in non-treated patients (*p* = 0.84 and 0.26, respectively, Wilcoxon signed-ranks tests) (Table 2). Although “definite CNV” group tended to show worsening of functional and structural parameters, the differences were not statistically significant (ETDRS, *p* = 0.18, CFT, *p* = 0.34, Wilcoxon signed-ranks tests). Patients’ VA and retinal thickness measurements either remained unchanged or worsened at the one-year follow-up visit, with none of the patients showing signs of improvement. In the “no CNV” group, 55% of patients showed spontaneous improvement (Table 2). However, mean ETDRS and CFT measurements at the one-year point were not significantly different from baseline measurements (*p* = 0.52 and 0.25, respectively).

Within treated patients, patients who underwent monthly injections received a significantly higher number of injections within the one-year follow-up period compared to the PRN group (mean ± SD, 6.5 ± 2.2 and 2.8 ± 2.2, respectively, *p* < 0.001, Mann–Whitney U test). However, the responses to either anti-VEGF treatment regimens were statistically equivalent. A monthly injection regimen was not superior to the PRN regimen in terms of the one-year changes in ETDRS VA or CFT (*p* = 0.47 and 0.42, respectively), or in the frequency of anatomical/functional improvement (*p* = 0.67, Chi-square test).

### 3.5. Comparison between Groups

**Figure 4:**
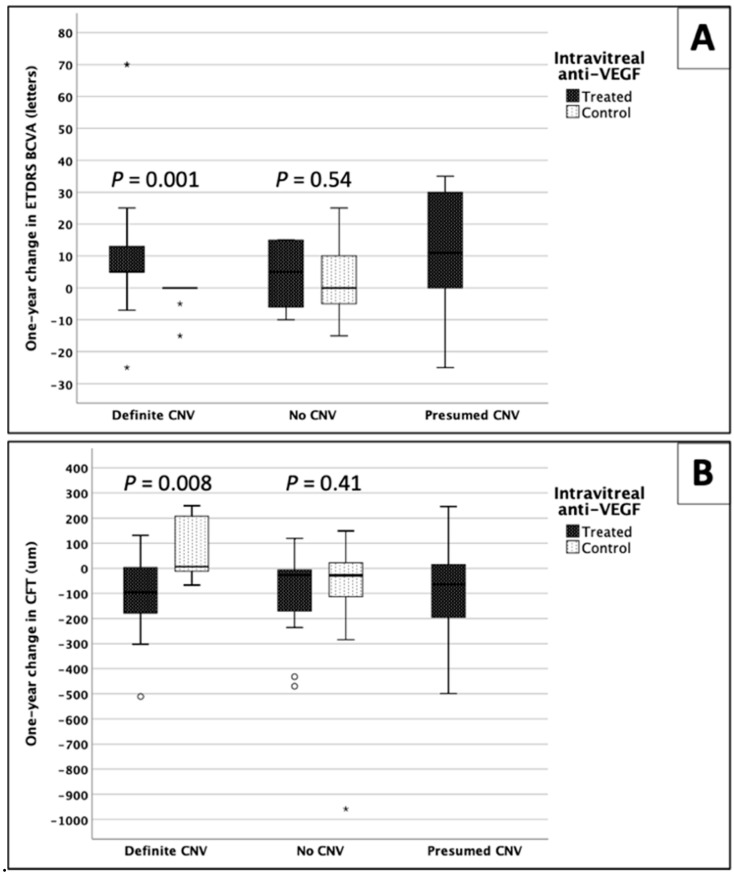
Box and whisker plots demonstrating one-year changes in (**A**) visual acuity (VA) and (**B**) central foveal thickness (CFT) in treated patients and control non-treated subjects. Circle and asterisk represent outliers. * *p* values are based on Mann–Whitney U test. ETDRS, early treatment diabetic retinopathy study; BCVA, best corrected VA; anti-VEGF, anti-endothelial growth factor; CNV: choroidal neovascularization.

Bevacizumab-treated CSCR patients with “definite CNV” improved significantly as compared to non-treated patients (*p* = 0.001 and 0.008 for ETDRS and CFT, respectively, Mann–Whitney U tests) (Table 3 and Figure 4). In contrast, anti-VEGF treatment did not cause significant changes in patients with no evidence of CNV on OCTA. Mean difference in VA and CFT over the one-year period was comparable between treated and non-treated “no CNV” groups (*p* = 0.54 and 0.41, respectively, Mann–Whitney U test). One-year changes in the treated “presumed CNV” group were not significantly different from other treated groups (Table 3).

The frequency of functional and structural improvement was statistically different among the five groups (*p* = 0.001 and *p* = 0.02, respectively, Pearson’s Chi-Square test) (Table 3). Post hoc analysis revealed most improvement was in the treated CSCR-CNV group of patients (z-score = 2.7 and 2.0 for ETDRS and CFT, respectively), while the least improvement was in the non-treated CSCR-CNV patients (z-score = −3.5 and −2.9, respectively). The frequencies of VA and CFT improvement in the other three groups were not statistically significant (|z-score| ≤ 1.2) (Table 3).

### 3.6. Predictive Factors of Response to Anti-VEGF Treatment

We performed univariable logistic regression analyses to investigate potential predictive biomarkers for anatomical or functional improvement in patients with suspected neovascular CSCR after intravitreal bevacizumab. Hyperreflective SIRE, SRF, SHRM and CNV on OCTA were significantly associated with a positive treatment response (*p* < 0.05). For the multivariable analysis, we included age, gender, hyperreflective SIRE, SRF, IHRF, SHRM and CNV (*p* < 0.2, univariable logistic regression). However, none of these variables proved to be independently associated with disease response to treatment.

## 4. Discussion

In this study, the response to intravitreal bevacizumab injections was retrospectively investigated over a one-year period in a cohort of chronic CSCR patients with suspected secondary CNV. Our findings revealed significant functional and anatomical improvement in OCTA-confirmed CNV secondary to CSCR after anti-VEGF injections, as compared to control non-treated patients. On the contrary, injections did not improve the one-year VA and CFT outcome in treated CSCR patients with no CNV on OCTA when compared to non-treated patients, defined by OCT evidence of CNV. In addition, OCTA showed significantly higher inter-grader reliability as compared to FA.

Intravitreal anti-VEGF has been the first-line treatment in suspected CNV secondary to CSCR [1]. The findings of this study confirmed that, and further revealed the limited efficacy of anti-VEGF in the absence of CNV, thus accentuating the importance of confirming presence of CNV on OCTA prior to treatment. Although FA and ICGA have been classically used to detect CNV, the identification of neovascularization in eyes with chronic CSCR can be challenging [22]. OCTA was proposed as an alternative for diagnosing CNV [23,24]. However, *en face* OCT angiograms need to be interpreted with caution. Areas with RPE and choriocapillaris abnormalities can cause more OCT signal to reach deeper choroidal layers (choroidal hypertransmission), resembling the appearance of neovascular membranes on the *en face* image (Figure 2). The key differentiating factor on B-scans is the presence of flow signal above Bruch’s membrane in CNV cases (Figure 2). In such cases, it is of great importance to distinguish “real” flow signal in the outer retina and sub-RPE space from projection artifacts arising from retinal vasculature (Figure 2) [25].

Multivariable logistic regression analysis identified SIRE with sub-RPE hyperreflectivity, as well as SHRM, as significant predictive factors for the presence of CNV on OCTA. SIRE or “double-layer sign” is described in the literature as long and shallow RPE elevation on OCT B-scans in AMD and pachychoroid spectrum [26,27,28,29,30,31]. In neovascular CSCR, CNV is likely to cause the elevation of RPE, with hyperreflectivity representing the fibrovascular tissue [31,32]. This should be discriminated from other forms of RPE detachments, including hyporeflective SIRE which is more likely to be seen in non-neovascular CSCR [31]. SHRM has been also previously described in CSCR patients [33], but this is the first paper to report its association with CNV in chronic CSCR. The exact composition of SHRM is not clear, but it was suggested to contain fibrin, exudation and/or fibrovascular tissue [34]. SHRM has been considered as a sign of activity in neovascular AMD, and it often resolves after anti-VEGF treatment with positive functional outcomes [35,36,37]. However, the prognostic value of SHRM in neovascular CSCR needs to be studied.

The vast majority of eyes with OCTA evidence of CNV showed anatomical or functional improvement after anti-VEGF. Meanwhile, more than 65% of the treated non-neovascular CSCR patients in our cohort also showed some degree of improvement. However, we believe that the improvement in this group of patients can be part of the natural history of non-neovascular CSCR [38] and does not necessarily represent response to intravitreal bevacizumab. This was confirmed by the similar pattern of spontaneous improvement that we observed in 55% of non-treated non-neovascular CSCR eyes. Although self-resolution with good functional and anatomical prognosis are typically expected in acute CSCR, the mean duration of the disease in the non-treated “no CNV” group was 5.3 years. Thus, prognosis in this group is expected not to be as good as in acute CSCR patients. In addition, a recent study by Lotery et al. reported a similar frequency (54%) of spontaneous SRF improvement in placebo-treated chronic CSCR patients [39]. In contrast, none of the non-treated “definite CNV” patients in our study showed improvement in VA or reduction in CFT at the one-year timepoint, and more than half of them showed significant worsening in VA or retinal thickness. Detailed natural history of this group of patients still needs to be studied. We also investigated a group of “presumed CNV” patients who were treated for suspected CNV based on OCT and FA, but did not have OCTA imaging to confirm the presence or absence of CNV. The rate of improvement after anti-VEGF among this group was slightly better than the “no CNV” group, but less than “definite CNV”. Although multimodal imaging is required for the diagnosis of CNV in CSCR, FA and OCT alone are often inadequate to provide a confirmatory diagnosis. We believe that OCTA should be used to confirm CNV and better stratify this group of patients.

Nevertheless, multivariable logistic regression analysis did not identify the presence of CNV, SIRE or SHRM as significant predictors of improvement after treatment. Since we analyzed all treated patients, including non-neovascular patients, the spontaneous bevacizumab-independent improvement can interfere with our analysis, underestimating the significance of anatomical biomarkers in predicting the response to anti-VEGF.

Our study included a cohort of CSCR patients with suspected secondary CNV. SD-OCT imaging is performed routinely for CSCR patients. Identifying biomarkers from OCT scans that potentially confer increased risk of the presence of CNV might be valuable for proper management. Detecting SHRM or SIRE with sub-RPE hyperreflectivity from OCT B-scans can be performed practically in the clinic to identify high-risk patients. However, our study shows that OCTA guided diagnosis of CNV is more reliable and the outcomes of anti-VEGF are better in the presence of CNV on OCTA. Thus, these findings suggest that OCTA should be performed in chronic CSCR eyes with a clinical/OCT suspicion of CNV, and anti-VEGF agents should ideally be initiated only for patients with an evidence of CNV on OCTA.

This paper provides valuable evidence on the role of OCTA in the diagnosis of CNV that responds to anti-VEGF in chronic CSCR. However, despite the large number of patients with chronic CSCR in this study, there were several limitations including the retrospective design and the relatively small categories. Since our data are based on real-life clinical data, treated and non-treated groups showed different clinical baseline features. Larger prospective controlled studies might be needed to confirm the findings of this study. Additionally, we only included patients receiving bevacizumab. Further studies are needed to investigate the efficacy of other anti-VEGF agents. Another limitation of this study was the lack of quantitative OCTA metrics in our analysis. Although qualitative assessment of OCTA can be more applicable in busy clinical settings, it also carries the risk for inter-grader variability in interpretation [37]. To avoid this, images were reviewed by two graders.

In conclusion, OCTA can be a valuable adjunct diagnostic tool for reliable confirmation of the presence of CNV in CSCR [24] and the CNV confirmed by OCTA responds well to bevacizumab injections. The response observed in presumed CNV is similar to the natural history of the disease, as noted in our control group with no CNV. OCTA is recommended in all CSCR eyes with suspected CNV as we observed that a proportion of our patients presumed to have no CNV on clinical examination, OCT and FA and left untreated had definitive CNV on OCTA and responded well to anti-VEGF. Larger and prospective studies are needed to confirm our findings.

## Figures and Tables

**Table 1:** Demographics and clinical data of study subjects.

	Bevacizumab-Treated			Non-Treated	
	Definite CNV	No CNV	Presumed CNV	Definite CNV	No CNV
**No. of participants**	22	15	22	9	20
**Gender**					
**Male**	9	10	13	5	17
**Female**	13	5	9	4	3
**No. of eyes**	23	15	23	9	22
**Anti-VEGF regimen**					
**3 Loading doses**	18	8	10	NA	NA
**1 dose + PRN**	5	7	13	NA	NA
**Age**	60.0 ± 10.9	54.1 ± 4.4	56.4 ± 14.5	64.3 ± 6.6	54.1 ± 12.0
**Range**	37–76 years	41–87 years	31–86 years	57–77 years	33–83 years
**Duration of CSCR**	6.4 ± 5.3	5.1 ± 4.4	6.2 ± 4.6	6.3 ± 4.6	5.3 ± 6.4
**Range**	2–21 years	0.16–15 years	0.42–12 years	1–9 years	0.17–20 years
**History of steroid use**	8	7	6	4	8
**Previous PDT**	4	0	2	0	1

Values are equal to N or population mean ± standard deviation (SD). CNV, choroidal neovascularization; anti-VEGF, anti-vascular endothelial growth factor; PRN, pro-re-nata; CSCR, central serous chorioretinopathy; PDT, photodynamic therapy; NA, not applicable.

**Table 2:** Functional and anatomical findings in included eyes.

	Bevacizumab-Treated			Non-Treated	
	Definite CNV	No CNV	Presumed CNV	Definite CNV	No CNV
**Central foveal thickness (CFT) (µm)**					
**Baseline**	379.0 ± 132.8	434.7 ± 157.8	432.6 ± 232.2	220.3 ± 78.2	315.7 ± 212.2
**1-Year**	277.2 ± 83.2	323.4 ± 105.4	357.0 ± 233.1	284.7 ± 114.4	228.3 ± 56.9
**Difference**	−98.5 ± 143.4	−110.9 ± 175.6	−86.3 ± 183.8	64.3 ± 122.3	−87.4 ± 223.6
**% Change**	−20.2 ± 29.1%	−19.6 ± 29.1%	−12.0 ± 49.7%	36.6 ± 61.4%	−10.7 ± 42.3%
***p* value**	0.03 *	0.14	0.24	0.43	0.25
**Frequency of improvement**	64%	46%	52%	0%	36%
**ETDRS BCVA (letters)**					
**Baseline**	58.7 ± 22.0	59.0 ± 18.2	40.7 ± 23.0	65.0 ± 13.2	65.6 ± 16.0
**1-Year**	70.3 ± 17.0	63.1 ± 21.9	50.9 ± 29.3	62.8 ± 13.7	66.9 ± 20.0
**Difference**	11.6 ± 20.8	3.6 ± 9.6	11.8 ± 17.2	−2.2 ± 5.1	1.7 ± 10.8
***p* value**	0.03 *	0.21	0.09	0.18	0.52
**Frequency of improvement**	78%	57%	62%	0%	43%
**Frequency of improvement** **in either CFT or VA**	96%	67%	70%	0%	55%
**Baseline OCT biomarkers**					
**SIRE**	86%	46%	83%	89%	45%
**Sub-RPE hyperreflectivity**	82%	23%	66%	89%	27%
**Sub-RPE hyporeflectivity**	4%	23%	17%	0%	18%
**Subretinal fluid**	91%	77%	91%	67%	86%
**Intraretinal hyperreflective foci**	55%	46%	61%	11%	23%
**Subretinal hyperreflective foci**	82%	85%	87%	44%	82%
**Subretinal hyperreflective material**	77%	62%	87%	33%	23%
**Intraretinal cysts**	14%	54%	65%	22%	9%
**Pachychoroid**	91%	92%	87%	89%	100%

Values are equal to population mean ± standard deviation (SD) or percentage. *p* values are based on Wilcoxon signed-ranks test and are adjusted for multiple comparisons using Holm–Bonferroni correction. * *p* values < 0.05 are marked with asterisk. CNV, choroidal neovascularization; ETDRS, early treatment diabetic retinopathy study; BCVA, best corrected visual acuity; OCT, optical coherence tomography; RPE, retinal pigment epithelium; SIRE, shallow irregular RPE elevation.

**Table 3:** Comparison between one-year changes among patient groups.

	ETDRS VA	CFT
**Mean change after 1 year (*p* value, Mann–Whitney U test)**		
**Treated “definite CNV” vs. non-treated “definite CNV”**	0.001 *	0.008 *
**Treated “no CNV” vs. non-treated “no CNV”**	0.54	0.41
**Treated “presumed CNV” vs. treated “no CNV”**	0.13	0.99
**Treated “presumed CNV” vs. treated “definite CNV”**	0.59	0.79
**Frequency of improvement (*p* value, Pearson’s Chi-square test; and z score)**		
**All groups**	0.001 *	0.021 *
**Treated “definite CNV”**	2.7 *	2.0 *
**Treated “no CNV”**	0.2	0.1
**Treated “presumed CNV”**	0.8	0.8
**Non-treated “definite CNV”**	−3.5 *	−2.9 *
**Non-treated “no CNV”**	−1.2	−0.9

*p* values < 0.05 and |z score| > 1.96 are marked with asterisk. ETDRS, early treatment diabetic retinopathy study; VA, visual acuity; CFT, central foveal thickness. Z score is based on adjusted standardized residuals from Chi-square test.
